# Hypocretin role in posttraumatic stress disorder-like behaviors induced by a novel stress protocol in mice

**DOI:** 10.3389/fpsyt.2023.1196994

**Published:** 2023-06-29

**Authors:** Tung-Yen Lee, Pei-Lu Yi, Fang-Chia Chang

**Affiliations:** ^1^Graduate Institute of Brain and Mind Sciences, College of Medicine, National Taiwan University, Taipei, Taiwan; ^2^Department of Sport Management, College of Tourism, Leisure and Sports, Aletheia University, Taipei, Taiwan; ^3^Department of Veterinary Medicine, School of Veterinary Medicine, National Taiwan University, Taipei, Taiwan; ^4^Neurobiology and Cognitive Science Center, National Taiwan University, Taipei, Taiwan; ^5^Graduate Institute of Acupuncture Science, College of Chinese Medicine, China Medical University, Taichung City, Taiwan; ^6^Department of Medicine, College of Medicine, China Medical University, Taichung City, Taiwan

**Keywords:** amygdala, anxiety, fear memory, lateral hypothalamic area (LHA), multiple prolonged stress (MPS), hypocretin, PTSD

## Abstract

**Introduction:**

Posttraumatic stress disorder (PTSD) is a psychiatric disorder developed in individuals who expose to traumatic events. These patients may experience symptoms, such as recurrent unwanted memory of the traumatic event, avoidance of reminders of the trauma, increased arousal, and cognitive difficulty. The hypocretinergic system originates from the lateral hypothalamic area (LHA) and projects diffusely to the whole brain, and hypocretin may be involved in the features of stress-related disorder, PTSD.

**Methods:**

Our study aimed to investigate the role of basolateral amygdala (BLA) hypocretin signals in the pathophysiology of PTSD-like symptoms induced by the modified multiple-prolonged stress (MPS) protocol. The BLA, a brain region involved in fear-related behaviors, receives the hypocretin projections. In this study, TCS1102, a dual hypocretin receptor antagonist, was used to block the hypocretin signal in BLA.

**Results:**

Our data indicated that the MPS protocol is a potential PTSD-like paradigm in mice. Meanwhile, the blockade of hypocretin signaling in the BLA relieved the MPS-induced fear response, and partially reduced PTSD-like anxiety behaviors performed by the open field test (OFT) and elevated plus maze (EPM) task.

**Discussion:**

Our findings suggest that the hypocretinergic system is a potential therapeutic approach for PTSD treatment. With further research, the hypocretin-based medication can be a candidate for human PTSD treatment.

## 1. Introduction

### 1.1. Post-traumatic stress disorder (PTSD)

PTSD is a psychiatric disorder that disturbs someone who has experienced or witnessed one or more traumatic events (1, 2). Traumatic events in humans include accidents, abuse, disasters, terrorist attacks, etc. (1). After experiencing traumatic events, four major categories of clinical symptoms may appear in patients with PTSD (1, 2). These symptoms include intrusion, avoidance, arousal and reactivity alteration, and cognitive and emotional symptoms (1, 2) as follows:

1. Intrusion symptoms: The intrusion symptoms are known as “re-experiencing” symptoms (3, 4). Patients repeatedly experience memories, dream of the traumatic event, or experience unpleasant thoughts related to the event (1, 5). Meanwhile, the patient may also have stress responses, such as sympathetic nervous system activation (3, 6). Clinically, intrusion symptom is a specific clinical manifestation of PTSD and helps distinguish PTSD from other trauma-related psychiatric disorders (1, 3).
2. Avoidance symptoms: The patients may avoid places or objects associated with the traumatic event (1, 2). In addition to avoidant behaviors, patients may also exhibit avoidant thoughts or feelings (2).
3. Alterations of arousal and reactivity: Patients with PTSD are more likely to be startled, nervous, or irritable than people without PTSD (1, 2). In addition, these patients also have difficulty concentrating, falling asleep, or staying asleep (1, 7, 8).
4. Cognitive and emotional symptoms: Patients may have difficulty remembering key features of events in terms of cognitive function (1, 2). At the emotional level, there will be self-blame, negative emotions, or loss of interest in past activities.

According to the Diagnostic and Statistical Manual of Mental Disorders, Fifth Edition (DSM-5), PTSD patients are 80% more likely than non-PTSD patients to have clinical symptoms that meet the diagnostic criteria for at least one of the other psychiatric disorders (1, 9). Common psychiatric comorbidities include anxiety, depression, bipolar disorder, or substance use disorder (9).

From the perspective of public health and epidemiology, although the incidence of PTSD is lower than other common mental diseases (10), PTSD is an important issue in modern mental health research because of the high incidence of comorbidities of PTSD.

### 1.2. Modifications of the multiple prolonged stress protocol and the potential application in mice

Several models have been developed and used to understand the underlying biological basis of PTSD. The current induction methods of PTSD-like behaviors in rats mainly include restraint, foot shock, and single prolonged stress (SPS) (11–15). Rats exposed to these stressors exhibited PTSD-like behaviors in humans, such as altered concentrations of stress hormones, the elevation of fear memory responses, increased anxiety levels, and sleep disturbances (15, 16). However, most of the PTSD-like behaviors induced by these models only last for about one to 4 weeks (13).

To overcome the relatively short-lasting PTSD-like behaviors from the original animal models, we employed the SPS protocol as a template and modified it to increase the stress intensity and unpredictability by increasing the frequency of stressor exposures and randomizing the orders of stressor administrations.

With this modification, the lasting duration of PTSD-like behaviors in rats has been extended to seven to eight weeks, and the corticosterone concentrations, fear memory behaviors, anxiety levels, and sleep behavior changes were similar to the manifestations observed in human PTSD patients (Yun Lo, unpublished data). We named this novel model “the multiple-prolonged stress (MPS) protocol.”

A previous report reveals that the mice may require higher stress intensity to induce PTSD-like behaviors (14). Meanwhile, the inconsistency of stress-related models in mice has been reported (15, 17, 18). Based on the success of long-lasting behavioral alterations induced by the MPS protocol in rats, we applied this protocol to mice to overcome the research dilemma of insignificant PTSD-like behaviors in mice. With the application of the MPS protocol in mice, we investigated the neuro-functional projections of PTSD-like behaviors to discover the possible pathophysiological mechanisms of PTSD.

### 1.3. The role of the amygdala in fear memory

Animal studies from the past decades reveal that the amygdala is an important brain region contributing to fear memory formation (19–22). The functional anatomy of the amygdala indicates that sensory information is delivered into the amygdala through two pathways (22). One is the direct pathway, which delivers the information directly from the thalamus to the amygdala; while the other one is the indirect pathway, in which the information is sent to the sensory cortex, and then relayed to the amygdala. Both pathways contribute to the process of fear conditioning (22).

Human studies also demonstrate that the amygdala plays an essential role in fear conditioning (22). In the bilateral amygdala lesions, the patient showed a dissociation response to exotic noxious stimuli (22). Meanwhile, fMRI studies also indicate that the activation and connectivity of the amygdala are related to PTSD (23, 24).

### 1.4. PTSD and the hypocretinergic system

Hypocretin, also known as orexin, is a neuropeptide that plays a crucial role in regulating various physiological processes, including stress response, emotional memory formation, sleep–wake cycles, appetite, arousal, reward, and thermoregulation (25–28). It is primarily produced in the lateral hypothalamic area (LHA) (26), a region of the brain involved in controlling numerous bodily functions. Dysregulation of hypocretin signaling has been implicated in several psychiatric and neurological disorders, including PTSD (29). Research into hypocretin and PTSD aims to investigate the role of hypocretin in the pathophysiology of the disorder. Studies have shown that hypocretin neurons and receptors are involved in the stress response (26) and play a significant role in modulating emotional and fear-related behaviors (28). Dysregulation of the hypocretin system has been observed in individuals with PTSD, suggesting its potential involvement in the development and maintenance of the disorder. By studying hypocretin in the context of PTSD, researchers seek to unravel the specific mechanisms by which hypocretin influences the symptoms and neurobiology of the disorder. This knowledge can lead to the development of targeted therapies that modulate hypocretin signaling to alleviate PTSD symptoms. It may also contribute to a deeper understanding of the broader neurobiological processes underlying stress-related psychiatric disorders, potentially paving the way for more effective treatments in the future.

One of the major targets of hypocretin projections is the brainstem, particularly the locus coeruleus and the dorsal raphe nucleus (25). The locus coeruleus is a small nucleus in the brainstem involved in regulating arousal, attention, and stress responses. The dorsal raphe nucleus is another brainstem region involved in modulating mood, sleep, and pain processing (25). Additionally, hypocretin neurons send projections to various other regions of the brain, including the amygdala, hippocampus, basal forebrain, and cerebral cortex. These areas play crucial roles in emotion regulation, memory formation, and cognitive processes (25, 26, 28). The basolateral amygdala (BLA), which is an important part of the fear circuit, receives the signal from the hypocretinergic system (29, 30). Due to the relation between the hypocretin function and PTSD manifestations, we would investigate the role of the LH-BLA circuit in PTSD-like behaviors. If the hypocretin signal in the BLA was blocked, we might be able to observe the reduction in fear memory and anxiety levels. The hypocretin system consists of two neuropeptides: hypocretin-1 and hypocretin-2. Hypocretin acts on specific receptors, known as hypocretin receptor 1 (HCRTR1) and hypocretin receptor 2 (HCRTR2), which are widely distributed throughout the brain (25). Therefore, our pharmacological approach aims to block both the HCRTR1 and HCRTR2 with the dual hypocretin receptor antagonist, TCS1102, to examine our hypothesis.

## 2. Materials and methods

### 2.1. Animals

Male C57BL/6 mice (BioLASCO Taiwan Co., Ltd.) were recruited for the experiments. These mice arrived at the housing environment at six-week ages, ranging from 22 to 26 g. The first week after arrival was the habituation period. The housing environment was set in a 12-h:12-h light:dark cycle. Food and water supply were *ad libitum*. The following experiment protocol has been approved by the Institutional Animal Care and Use Committee (IACUC), and the approval number is NTU-110-EL-00156. The sample sizes (n) for the vehicle-control group (Ctrl_BLA_Veh), the vehicle-MPS group (MPS_BLA_Veh), and the TCS-MPS group (MPS_BLA_TCS) are 11, 7, and 10, respectively.

### 2.2. Stereotaxic surgery

Stereotaxic surgery was performed on mice after seven-week age. The mouse was intraperitoneally anesthetized by the Zoletil/xylazine (43/6.364 mg/kg) combination. After the mouse was under anesthesia, the mouse’s head was fixed in the stereotaxic instrument, and povidone-iodine and 70% ethanol were used for disinfection. A 1-cm incision along the midline would be made to expose the bregma and lambda on the skull. The tips of microinjection cannulaes (C315GS, 26GA; Plastics One, United States) were placed in the bilateral BLA (AP: −1.8; ML: ±3.0; DV: −4.5). Two anchor screws were planted on the right frontal skull and left parietal skull with the dental acrylic (Tempron, GC Co., Tokyo, Japan) to assist the fixation of cannulaes. After the headpiece was made, neomycin ointment (Genuine Chemical Pharmaceutical Co., Ltd.) was given to the surgical area. After the neomycin administration, the mouse was kept in an individual acrylic cage for a one-week recovery. During the recovery period, ibuprofen (140 mg, Yung Shin Pharmaceutical Industrial Co., Ltd) was added to the drinking water for analgesia.

### 2.3. The multiple prolonged stress (MPS) protocol

**Figure 1:**
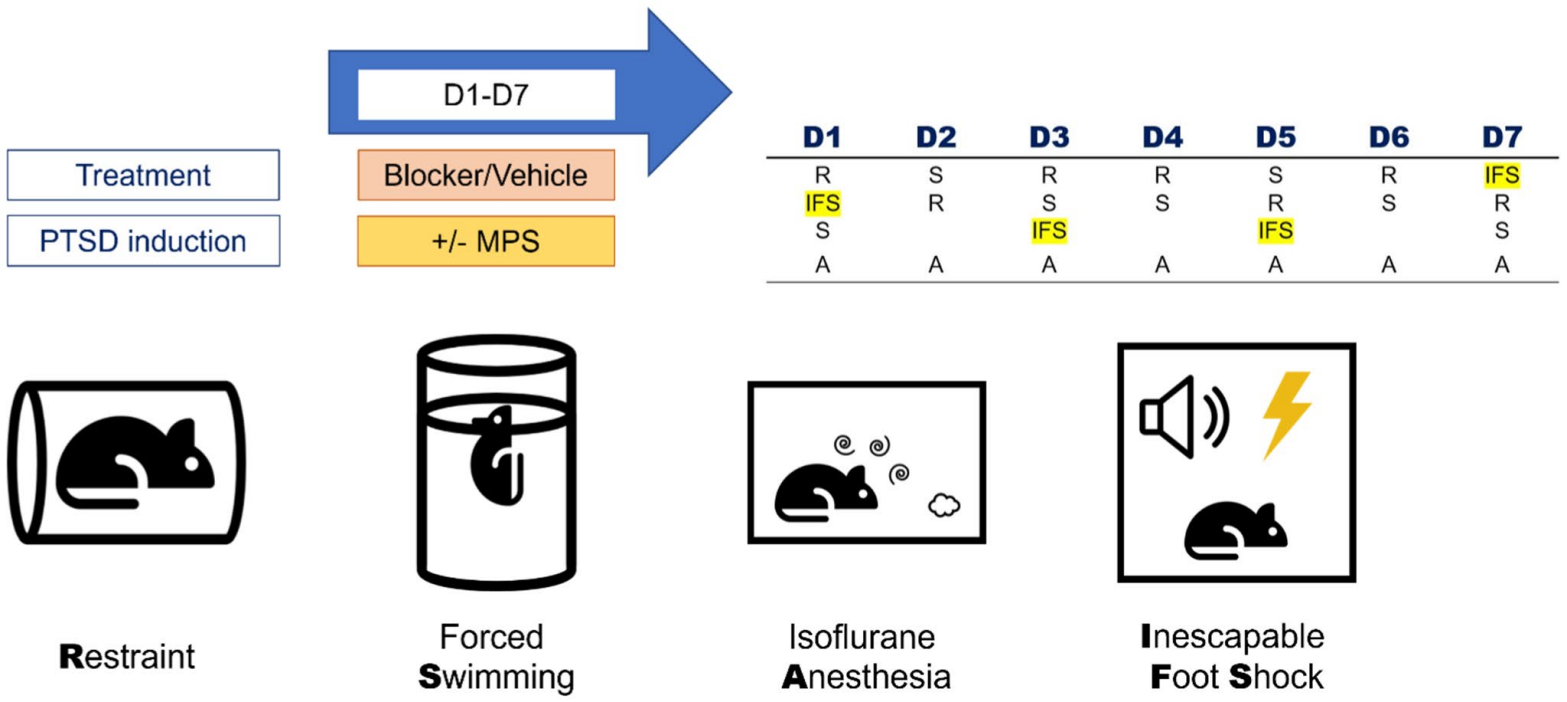
Components and daily manipulation orders of the MPS protocol. The MPS protocol contained four stressors: restraint (R), forced swimming (S), isoflurane anesthesia (A), and inescapable foot shock (IFS). The daily manipulation orders of the MPS protocol were listed in the right-upper table. Rat, thunder, cylinder, speaker, spiral and clouds icons are made by Freepik, Smashicons, Pixel perfect, Pixel perfect, riajulislam and juicy_fish from www.flaticon.com.

The MPS protocol was used to induce PTSD-like behaviors in mice. This protocol contained four stressors: restraint, forced swimming, isoflurane anesthesia, and inescapable footshock (Figure 1).

#### 2.3.1. Restraint

Polyethylene (PE) plastic bags (19 cm × 12 cm) with 40–60 ventilation pores made by a 24-Gauge needle were used as restrainers in the MPS protocol. The mouse was first put into the bag, and the operator would pinch the bag 0.5 cm above the mouse and roll up the mouse. The tape was used to fix the bag to achieve the effect of restraint. The “fixed roll” was put into a transparent plastic box with a steel net on the top to prevent the mouse from escaping the experimental environment. The whole duration of a restraint procedure was 2 hours.

#### 2.3.2. Forced swimming

A two-liter (L) glass beaker (Pyrex, Germany) containing 1.6 L of water was used as the forced swimming environment for a single mouse. The glass above the water level was 5 cm, which was high enough to prevent the mouse from escaping; meanwhile, the depth was 14.5 cm, which was deep enough to prevent the hindlimbs of a mouse from touching the ground. The maximum duration of a forced swimming procedure was 20 minutes. The forced swimming was terminated once the nose tip of a mouse was below the water level.

#### 2.3.3. Isoflurane anesthesia

Isoflurane is a commonly used anesthetic in animals and humans and was used to replace the original anesthetic ether in the SPS model because of the potential explosive danger of ether. The 500 mL anesthesia chamber contained 2.5–5.0% isoflurane gas. The animal was put into the chamber for anesthesia, and the respiratory rate and the response to the external stimulus were monitored. Loss of consciousness in the experiment was defined as the respiratory decrease and the remaining unconsciousness after shaking the camber. Once the animal lost consciousness, the operator removed the animal from the chamber and sent it back to its home cage for recovery.

#### 2.3.4. Inescapable footshock (IFS)

The inescapable footshock was performed in an acrylic foot cage (L: 28 cm, W: 30 cm, H: 45 cm). A glass petri dish with 2% and 50 mL vinegar placed under the footshock grill was served as the contextual cue. In the MPS protocol, inescapable footshocks were performed on day 1, day 3, day 5, and day 7. During the 10-min inescapable footshock stimulation, a one-second, 80 decibels, and 2 k hertz cue tone was played before every single footshock stimulus. After the cue tone was played, the animal received a one-second and 0.5 milli-ampere of footshock stimulation. Within the whole inescapable footshock period, the animal received 12 randomized electrical shocks within a ten-minute duration.

#### 2.3.5. Fear memory retrieval

The cage used for fear memory retrieval is the same as that of inescapable footshock. The difference between these two conditions was that the footshock grill did not deliver the electrical stimulation. When examining the fear memory retrieval, the computer played 12 cue tones with 2 k hertz, 1 second, and 80 decibels; meanwhile, the behavior of the animal was filmed with the video system for analysis. The video analysis was performed with EthoVision XT14 (Noldus, The Netherlands). Freezing behavior is a commonly observed behavior in fear memory retrieval. The definition of freezing behavior in our experiment was that the tested animals showed no moving or other action for longer than 0.75-s duration, except for breathing (31). In this experiment, our assessments were categorized into three major types: the cue freezing response, the whole trial freezing response, and the whole trial inactive state. In the cue freezing response, a successful reaction to the cue tone was defined as the tested animals showing freezing behavior within the 1-s cue tone and the 4-s following time window. After counting the total frequency of cue tone response, the reaction rate was divided by twelve and transformed into the percentile. The cumulative duration of freezing behaviors would be calculated and performed in seconds.

In the whole trial freezing response, once the mouse behavior had met the criteria of freezing behavior in a ten-minute trial, the frequency and duration were counted. The frequency was performed in times, and the cumulative duration was depicted in seconds.

In the whole trial inactive state, once the mouse showed an inactive state, whether it met the criteria of freezing behavior or not, the duration was counted. The frequency was performed in times, and the cumulative duration was performed in seconds.

### 2.4. Anxiety-like behavior tests

The open field test and the elevated plus maze test were recruited to assess the anxiety level.

#### 2.4.1. Open field test (OFT)

The open field test is a commonly used behavior task in evaluating rodent anxiety-like behavior. This test is based on the conflict of two natures in mice, the nature of exploring novel environments, and the nature of escaping from light and predators. The conflict between the two natures leads to the elevation of anxiety.

The testing maze of the open field test was a 40 × 40 × 40 cm^3^ non-covered white-matte acrylic box. The bottom surface of the box was 40 × 40 cm^2^, with a 20 × 20 cm^2^ square marked in the central part of the surface as the center zone in the open field.

When the open field test started, the tested mouse was placed into the testing field for 10 minutes. Within 10 minutes, the tested animal could freely travel around the field. The animal behavior was captured by the camera system installed on the roof of the behavior testing room. EthoVision XT14 (Noldus, The Netherlands) was used for the behavior analysis.

In the open field test, velocity, inner zone entry frequency, and inner zone cumulative duration were quantified. The velocity represents the locomotor activity of the tested animal during the test. Meanwhile, the entry frequency and cumulative duration of a tested animal staying within the central zone were used as the indicator for the anxiety level. The longer time stayed in the central zone, the lower the anxiety level the animal is.

#### 2.4.2. Elevated plus maze (EPM) task

Elevated-plus maze task is another commonly used behavior task for evaluating rodent anxiety levels. The task is based on the conflict of two natures in mice, the nature of exploring novel environments, and the nature of staying away from height and open environments. The conflict between the two natures elevates the anxiety level of the tested animal.

The elevated-plus maze in use is divided into five major parts: two open arms, two closed arms, and a square central area. This EPM was placed 42.5 cm above the floor. The size of each open arm or closed is 35 cm in length, and 6 cm in width, and the size of the central area is 6 × 6 cm^2^. The location of the open arms is opposite each other; the closed arms are in the same condition.

When the elevated-plus maze started, the tested mouse was placed into the central area of the maze, and let the animal freely explore among open arms, closed arms, and the central area for 5 minutes. The exploration process was recorded by the video system on the roof. If the mouse completed the task without falling from the maze, the video would be analyzed with EthoVision XT14 (Noldus, The Netherlands); if the animal fell from the maze, the animal would be ruled out from the task.

Velocity, entry frequency, and cumulative duration of the open arms/closed arms were quantified. The velocity represented the locomotor activity of the tested animal. The cumulative duration and entry frequency of a tested animal staying within open arms and closed zones were used as indicators for the anxiety level. The longer duration or higher entry frequency of the open arms represented the lower the anxiety level the animal would be; the longer duration or higher entry frequency of the closed arms alternatively represented the higher the anxiety level of the animal.

### 2.5. Body weight change

Some reports reveal that PTSD may increase the possibility of body weight change, either in body weight loss (32) or body weight gain (33). To understand how the MPS influences body weight changes in mice, the body weight data of each mouse would be daily recorded. In comparisons of body weight change, the whole trial was divided into three time periods: the whole trial (Day 1–Day 14), the PTSD-induction period (Day 1–Day 7), and the behavior testing period (Day 8–Day 14).

### 2.6. Drug preparation

The dual hypocretin receptor antagonist we used was TCS1102 (Adooq, United States). This compound is not water-soluble. Therefore, we used 20% Vitamin E TPGS (Sigma-Aldrich, United States) in pyrogen-free saline (PFS) as a vehicle. The final concentration of TCS1102 used in the experiment was 4 μg/μL.

To reveal the potential effects of BLA hypocretin signal in the fear memory acquisition and PTSD-like behaviors of MPS-exposed mice, the TCS1102 would be administrated before the MPS manipulation from day 1 to day 7.

### 2.7. Experimental designs

**Figure 2:**
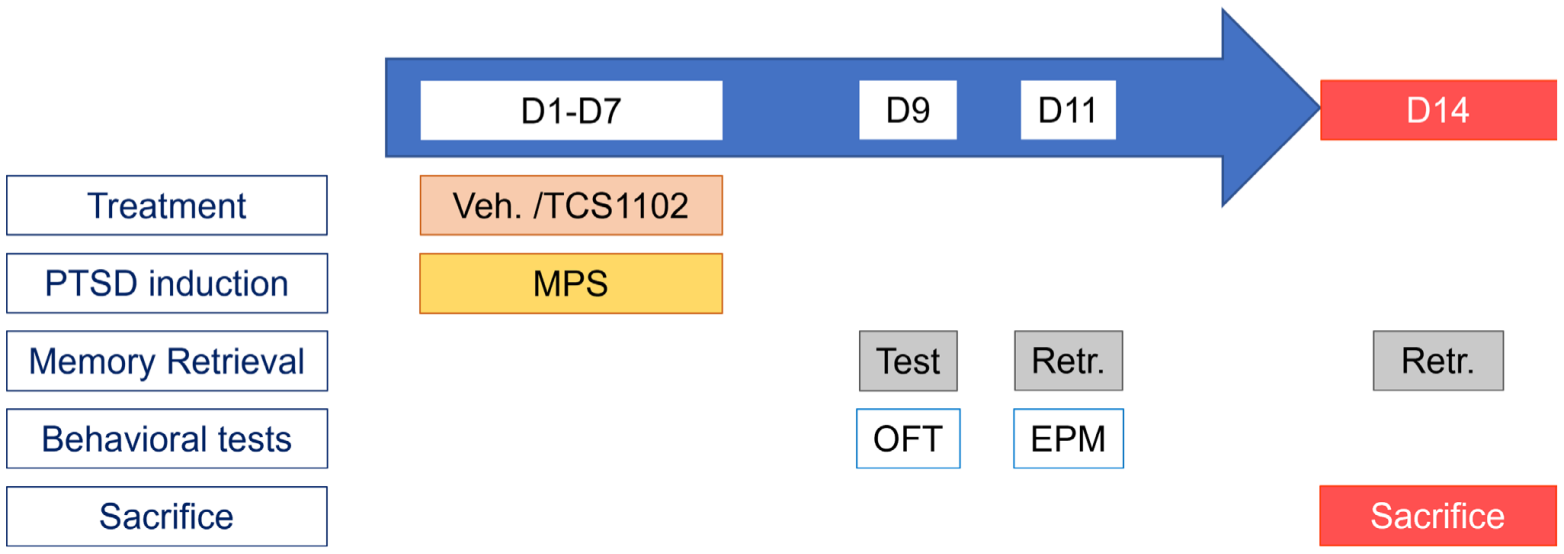
Protocol for studying the role of BLA hypocretin signal in PTSD-like behaviors. The experimental protocol can be divided into two parts: the PTSD induction period (D1–D7), and the behavior testing period (D8–D14). During the PTSD induction period, treatments (20% Vitamin E TPGS or TCS1102) were administrated before the MPS manipulation. During the behavior testing period, memory retrieval was evaluated on day 9, the open field test was performed on day 9, and the elevated plus maze was performed on day 11.

There were two goals in this experiment: the first one was to evaluate the effects of the MPS protocol in mice; the second was to evaluate the role of BLA hypocretin signal in the mice PTSD by pharmacological blockade. Figure 2 depicts the MPS protocol performed in mice.

The experimental duration was 14 days. The MPS protocol was performed in the first week to induce PTSD-like behaviors in mice. After the expression of PTSD-like behaviors, the open field test and the elevated plus maze task were used to assess the anxiety levels in the second week. Body weight would be recorded as an indication of the body condition.

The PTSD induction period was from day 1 to day 7. A total of 0.2 μL substance (either 20% Vitamin E TPGS, or TCS1102) was injected into the bilateral BLA before daily manipulations. The dose of TCS1102 was determined from our previous study (34). The MPS procedure for each day was shown in Figure 2.

The behavior testing period was from day 8 to day 14. Anxiety-like behavior tests were performed on day 9 and day 11. On day 9, the animal would first receive a ten-minute fear memory retrieval. After the evaluation, the animal was sent to the open field test. On day 11, the animal received the fear memory retrieval and was sent to the elevated plus-maze task. Our preliminary study indicated that the immobility responses are significantly elevated during the short-term (one to two weeks) and long-term (6 weeks) period after the memory retrieval in the MPS recollection from rats (unpublished data). In this study, we mainly focused on the role of hypocretin in BLA during short-term fear memory retrieval in mice. Therefore, the behavioral test period was set from day 8 to day 14.

Day 14 was the end of the experiment. On day 14, the animal first received the fear memory retrieval. After the retrieval, the animal was sent back to its homecage for a 90-min rest. By the end of the rest, the animal was euthanized with carbon dioxide at a 30% chamber replacement rate.

### 2.8. Statistics

The statistical analysis was performed by Prism 7 (GraphPad). One-way ANOVA followed by Dunnett’s multiple comparison was used for fear memory evaluations and the open field test. The Kruskal-Wallis test followed by Dunnett’s multiple comparison was used for the elevated plus maze task analysis. Two-way ANOVA followed by Dunnett’s multiple comparison was used for body weight change. In the multiple comparison phases, the mean rank of each group was compared with the MPS group. The significance level was set as 0.05 (*p* ≦ 0.05). The data were shown as “mean ± standard error (SEM)” in graphs.

## 3. Results

### 3.1. Fear memory evaluation

#### 3.1.1. Cue freezing response

**Figure 3:**
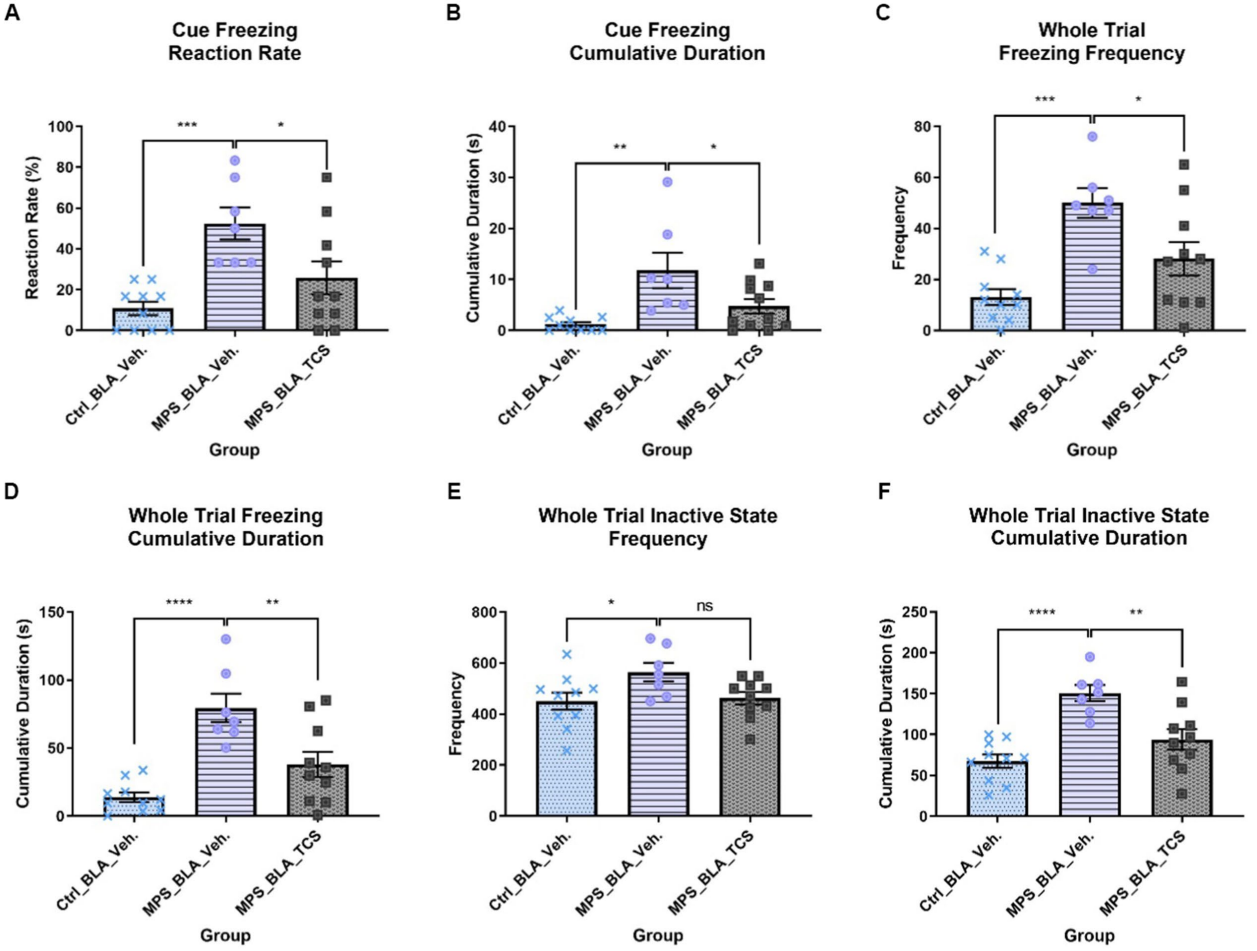
Blockade of hypocretin receptors in BLA reduced freezing behaviors induced by the MPS manipulation. The panels **(A–F)**, respectively, represent the values obtained from the cue freezing reaction rate, cue freezing cumulative duration, whole trial freezing frequency, the cumulative duration of whole trial freezing, the frequency of whole trial inactive state, and the cumulative duration of the whole trial inactive state. The sample sizes (*n*) for the vehicle-control group (Ctrl_BLA_Veh), the vehicle-MPS group (MPS_BLA_Veh), and the TCS-MPS group (MPS_BLA_TCS) are 10, 7, and 10, respectively. One animal in the Ctrl_BLA_Veh group was not included due to the video loss because of the recording system overheating. *: *p* < 0.05, **: *p* < 0.01, ***: *p* < 0.001; ns: non-significant.

The MPS manipulation induced significant cue-related fear responses in both the cue freezing reaction rate and cue freezing cumulative duration. The cue freezing reaction rate was elevated from 10.83 ± 3.299 in the control group treated with vehicle (the vehicle-control group, *n* = 10) to 52.38 ± 7.867 in the MPS manipulated group treated with vehicle (the vehicle-MPS group, *n* = 7) (*p* = 0.0006; Figure 3A), and the cue freezing cumulative duration was increased from 1.209 ± 0.4544 s in the vehicle-control group (*n* = 10) to 11.8 ± 3.468 s in the vehicle-MPS group (*n* = 7) (*p* = 0.0011; Figure 3B).

After the blockade of BLA hypocretin receptors by TCS1102, both the cue freezing reaction rate and cue freezing cumulative duration were reduced. The cue freezing reaction rate was reduced to 25.83 ± 8.094 (*n* = 10, vs. the vehicle-MPS group; *p* = 0.0221; Figure 3A) and the cue freezing cumulative duration was decreased to 4.734 ± 1.396 s (*n* = 10, vs. the vehicle-MPS group; *p* = 0.0227; Figure 3B).

#### 3.1.2. Whole trial freezing response

In the whole trial freezing response, the MPS manipulation induced significant fear response by increasing the whole trial freezing frequency (from 13.1 ± 3.16 in the vehicle-control group (n = 10) to 50 ± 5.79 in the vehicle-MPS group (*n* = 7); *p* = 0.0016; Figure 3C) and augmenting the cumulative duration of whole trial freezing response (from 13.76 ± 3.5 s in the vehicle-control group (*n* = 10) to 79.52 ± 10.58 s in the vehicle-MPS group (*n* = 7); *p* = 0.0004; Figure 3D).

Given the TCS1102 intro the BLA to block the hypocretin signals demonstrated similar relieving effects in both the whole trial freezing frequency and cumulative duration of the whole trial freezing response. The whole trial freezing frequency was reduced to 28.1 ± 6.528 (*n* = 10, vs. the vehicle-MPS group; *p* = 0.0190; Figure 3C), and the whole trial freezing response was decreased to 37.96 ± 9.288 s (*n* = 10, vs. the vehicle-MPS group; *p* = 0.0031; Figure 3D).

#### 3.1.3. Whole trial inactive state

MPS manipulation significantly increased the frequency of the whole trial inactive state from 450.5 ± 33.77 obtained after the vehicle-control group (*n* = 10) to 564.4 ± 36.4 (*n* = 7, *p* = 0.0387), while microinjection of TCS1102 into the BLA reduce the values to 462.7 ± 24.59 (*n* = 10) but it did not reach statistical significance (*p* = 0.0671; Figure 3E).

The MPS manipulation significantly increased the cumulative duration of the whole trial inactive state from 67.41 ± 8.069 s acquired from the vehicle-control group (*n* = 10) to 150.6 ± 9.906 s (*n* = 7, *p* < 0.0001; Figure 3F). Administration of TCS1102 significantly reduced the cumulative duration of the whole trial inactive state to 93.99 ± 12.54 s when compared with that of the vehicle-MPS group (*n* = 10, *p* = 0.0026; Figure 3F).

### 3.2. Anxiety-like behavior tests

#### 3.2.1. Open field fest (OFT)

**Figure 4:**
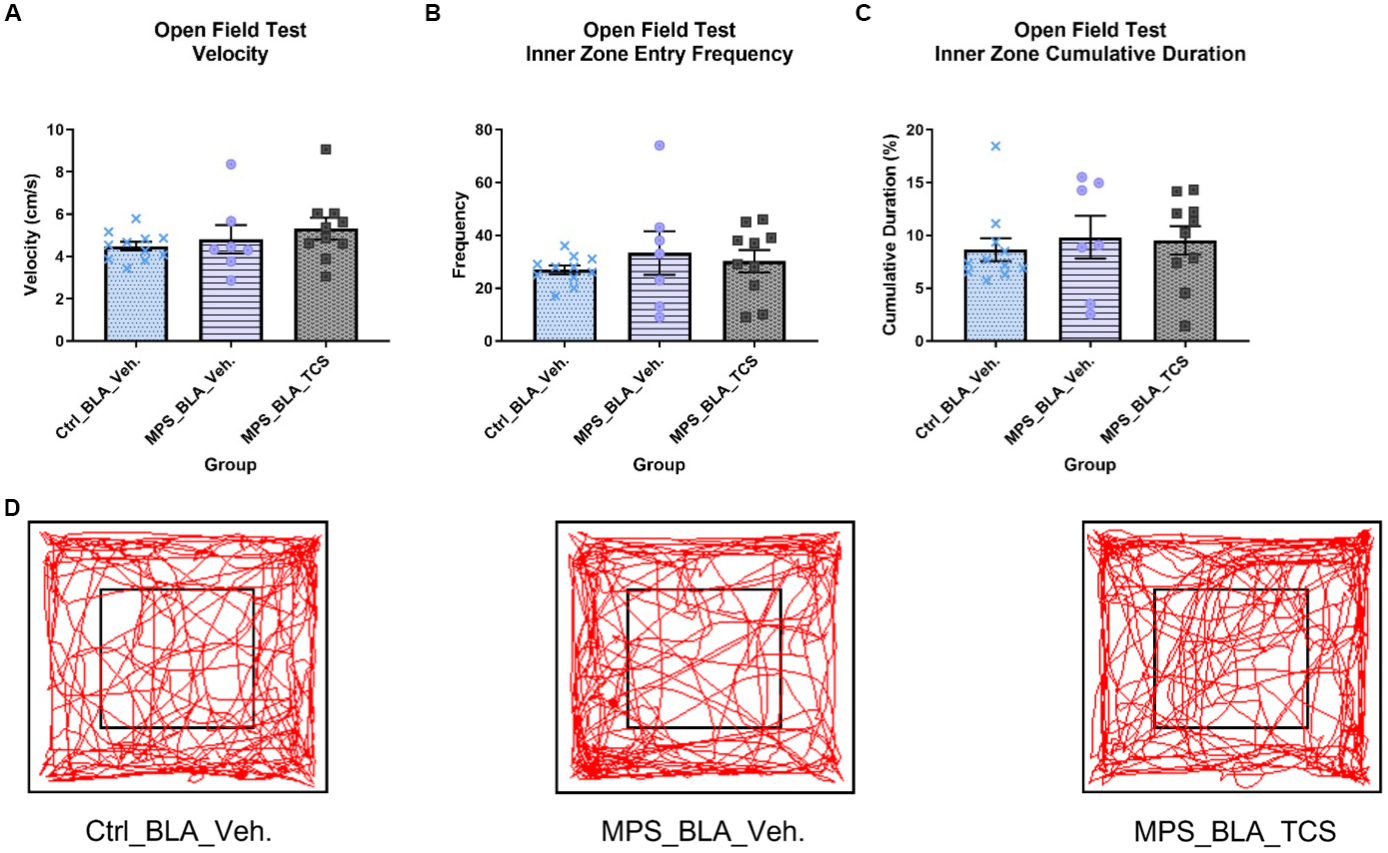
Blockade of hypocretin receptors in BLA did not change any parameters obtained from the OFT. The panels **(A–D)**, respectively, represent the values obtained from the velocity, inner zone entry frequency, the cumulative duration of the inner zone in the OFT, and the representative trace map for each group. The sample sizes (*n*) for the vehicle-control group (Ctrl_BAL_Veh), the vehicle-MPS group (MPS_BLA_Veh), and the TCS-MPS group (MPS_BLA_TCS) are 11, 7, and 10, respectively. Panel **(D)** represents the traces obtained from the OFT.

No statistically significant alteration in the velocity of OFT was found. The OFT velocities obtained in the vehicle-control group (*n* = 11), vehicle-MPS group (*n* = 7), and TCS-MPS group (*n* = 10) were 4.483 ± 0.2048, 4.819 ± 0.6702 (vs. vehicle-control group, *p* = 0.8218), and 5.318 ± 0.5113 (vs. vehicle-MPS group, *p* = 0.6689; Figure 4A).

The MPS manipulation showed no significant changes when comparing the inner zone entry frequency between the vehicle-MPS group (*n* = 7, 33.29 ± 8.283) and the vehicle-control group (*n* = 11, 26.91 ± 1.626, *p* = 0.5251). Blockade of hypocretin receptors in the BLA exhibited no change in the inner zone entry frequency (*n* = 10, 30.2 ± 4.218) when compared with that of the vehicle-MPS group (*p* = 0.8537; Figure 4B).

The MPS manipulation also demonstrated no significance when comparing the cumulative duration of the inner zone between the vehicle-MPS group (*n* = 7, 9.814 ± 2.028 s) and the vehicle-control group (*n* = 11, 8.638 ± 1.083 s, *p* = 0.7844). Blockade of hypocretin receptors in the BLA exhibited no change in the cumulative duration of the inner zone (*n* = 10, 9.549 ± 1.334 s) when compared with that of the vehicle-MPS group (*p* = 0.9876; Figures 4C,D).

#### 3.2.2. Elevated plus maze (EPM)

**Figure 5:**
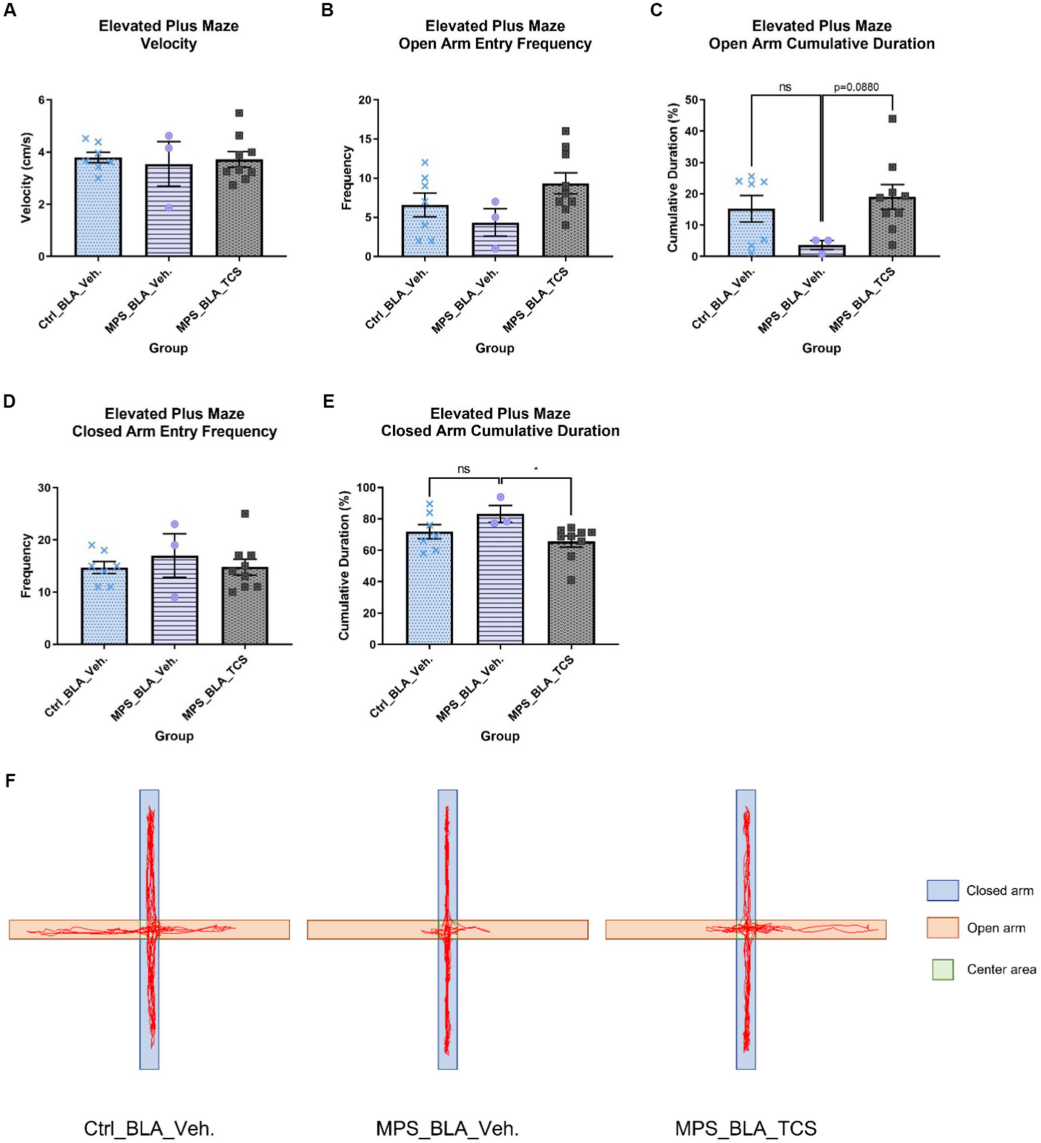
Blockade of BLA hypocretin receptors on the MPS-induced anxiety in the EPM task. The panels from **(A–F)**, respectively, represent the values obtained from the velocity, open arm entry frequency, cumulative duration of the open arm, closed arm entry frequency, cumulative duration of the closed arm, and the representative trace map for each group. In **(F)**, light orange areas represent open arms, while light blue areas represent closed arms. The sample sizes (*n*) for the vehicle-control group (Ctrl_BAL_Veh), the vehicle-MPS group (MPS_BLA_Veh), and the TCS-MPS group (MPS_BLA_TCS) are 7, 3, and 9, respectively. Four animals in the Ctrl_BLA_Veh group, four animals in the MPS_BLA_Veh group, and one animal in the MPS_BLA_TCS were excluded due to falling from the EPM apparatus. Panel **(E)** represents the traces acquired from the EPM.

There was no significant difference in the locomotion velocity of the EPM task when comparing the vehicle-control group (*n* = 7, 3.789 ± 0.2033), the vehicle-MPS group (*n* = 3, 3.543 ± 0.8525), and the TCS-MPS group (*n* = 9, 3.721 ± 0.2944; Figure 5A).

The MPS manipulation demonstrated no significant alteration in the open arm entry frequency in the EPM task; the values obtained from the vehicle-control group were 6.571 ± 1.51 (*n* = 7) and that acquired from the vehicle-MPS group was 4.333 ± 1.764 (*n* = 3, vs. vehicle-control group, *p* = 0.7099). Administration of TCS1102 into the BLA also did not change the open arm entry frequency (*n* = 9, 9.333 ± 1.354, vs. vehicle-MPS group, *p* = 0.1430; Figure 5B). Analysis of the cumulative duration of the open arm, there was still no difference between the vehicle-control group (*n* = 7, 15.22 ± 4.235 s) and the vehicle-MPS group (*n* = 3, 3.663 ± 1.457 s, *p* = 0.1812; Figures 5C,F), although MPS manipulation tended to decrease the cumulative duration of the open arm. Administration of TCS1102 into the BLA tended to increase the cumulative duration of the open arm (*n* = 9, 19.01 ± 3.923 s), but it did not reach statistical significance (*p* = 0.0880; Figure 5C).

The MPS manipulation and TCS1102 administration also demonstrated no significant alteration in the closed arm entry frequency of the EPM task. The values obtained from the vehicle-control group, the vehicle-MPS group, and the TCS-MPS group were 14.71 ± 1.169 (*n* = 7), 17 ± 4.163 (*n* = 3) and 14.78 ± 1.535 (*n* = 9) (Figure 5D). The MPS manipulation tended to increase the cumulative duration of the open arm in the EPM task (*n* = 3, 83.21 ± 5.403 s) when compared with that obtained from the vehicle-control group (*n* = 7, 71.88 ± 4.49 s, *p* = 0.1859; Figures 5E,F). However, administration of TCS1102 into the BLA significantly reduced the cumulative duration of the closed arm in the EPM task to 65.62 ± 3.553 s (*n* = 9, *p* = 0.0329, Figures 5E,F), suggesting the involvement of BLA hypocretin signal in the MPS-induced anxiety effect.

### 3.3. Body weight change

**Figure 6:**
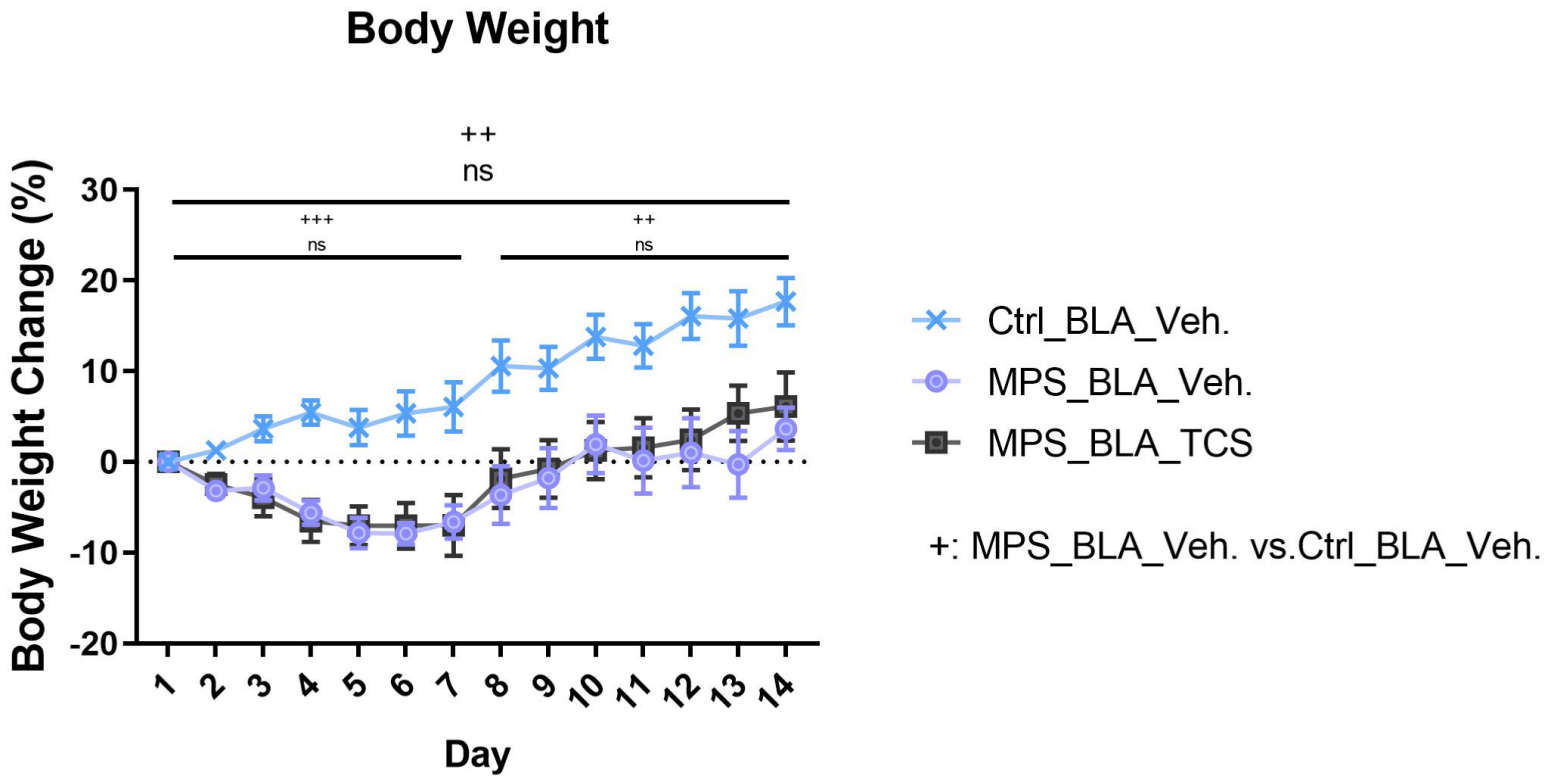
Blockade of hypocretin receptors in BLA did not change the body weight loss induced by the MPS protocol. The icon “+” represents the comparison between the vehicle-MPS group (MPS_BLA_Veh) and the vehicle-control group (Ctrl_BLA_Veh) group. The icon “ns” represents there was no significant difference between the vehicle-MPS group and the TCS-MPS group whether in the PTSD-induction, behavior testing periods, or the whole trial. The sample sizes (n) for the vehicle-control group (Ctrl_BAL_Veh), the vehicle-MPS group (MPS_BLA_Veh), and the TCS-MPS group (MPS_BLA_TCS) are 11, 7, and 10, respectively. ++: p < 0.01, +++: p < 0.001; ns: non-significant.

The MPS protocol significantly altered the percentage change in body weight during the whole trial. The body weight changes obtained from the vehicle-control (*n* = 11) and the vehicle-MPS group (*n* = 7) were 8.752 ± 1.562% and − 2.351 ± 0.9785%, respectively (*p* = 0.0027). However, there was no effect for TCS1102 to block the reduced body weight, and the percentage change of body weight is −1.419 ± 1.203 in the TCS-MPS group (*n* = 10, vs. vehicle-MPS group, *p* = 0.9382; Figure 6). Furthermore, the whole trial period could be divided into the MPS manipulation period and the behavioral test period. The MPS protocol reduced body weight during both periods, but TCS1102 did not rescue the body weight loss in both periods (Figure 6).

## 4. Discussion

PTSD is a mental health condition that can develop in individuals who have experienced or witnessed a traumatic event. The amygdala, a part of the brain that processes emotions, has been shown to play a significant role in PTSD (19, 20). Recent research has also highlighted the importance of the hypocretin system in the regulation of emotional responses (26, 28). Hypocretin, also known as orexin, is a neuropeptide that is produced in the hypothalamus and plays a crucial role in regulating wakefulness and sleep (25). The hypocretin system has also been implicated in the regulation of emotion, with studies suggesting that hypocretin neurons project to brain regions involved in emotion processing, including the amygdala (35, 36). The amygdala is a small, almond-shaped structure located deep in the brain’s temporal lobe. It is responsible for processing emotions, particularly fear and anxiety (19–21). In individuals with PTSD, the amygdala is often hyperactive, leading to exaggerated fear responses to stimuli that are not typically threatening (23). This hyperactivity has been linked to a reduced ability to inhibit fear responses and a decreased ability to regulate negative emotions (23). However, it is still unclear whether hypocretin in the BLA contributes to the fear memory formation in the establishment of PTSD. Therefore, in this study, we further explored the role of hypocretin and its receptors in the BLA by using pharmacological blockade in a PTSD mouse model.

In this study, we adapted a novel PTSD rat model, which has exhibited a long-lasting anxiety level after the MPS manipulation, to our current mouse model. Our previous study indicated that the freezing behaviors, anxiety levels, and the brain theta oscillations obtained from the local field potentials of BLA were enhanced and these enhancements could last for 60 days after applying the MPS protocol in rats (unpublished results). In the current study, our results indicated that the MPS manipulation also significantly increased freezing behavior during the retrieval in mice. The reaction rate, cumulative duration, and freezing frequency were significantly enhanced after the MPS manipulation, and the application of dual hypocretin receptor antagonist TCS1102 into the BLA blocked those enhancements. This result suggested the involvement of BLA hypocretin receptors in fear memory formation.

When analyzing anxiety-like behaviors from the OFT and EPM task, the MPS manipulation showed the anxiogenic trend but did not reach a statistically significant difference. However, the dual hypocretin receptor antagonist TCS1102 demonstrated a significant decrease in the cumulative duration of the closed arm during the EPM task, which indicated that blockade of BLA hypocretin signals exhibits the anxiolytic effect. Meanwhile, body weight loss could be observed during the PTSD-induction period, behavior testing period, and the whole trial. However, the inhibition of BLA hypocretin signals could not relieve the effect of body weight loss, suggesting that the stress-induced body weight loss was mediated by other systems rather than hypocretin.

Several studies have investigated the relationship between the hypocretin system and the amygdala in PTSD. One study found that hypocretin neurons project to the central nucleus of the amygdala (CeA), which is a key region involved in fear processing (37). This study also found that administration of hypocretin receptor 1 antagonist directly into the CeA or given by systemic route reduces the expression of conditioned fear (37), suggesting that the hypocretin projections to the CeA play a role in regulating the hyperactivity of the amygdala in individuals with PTSD. Since BLA also received the orexin innervations from the hypothalamus (29, 30), BLA possesses projections to the CeA (38). Our current study further depicted the role of BLA in the involvement of PTSD.

Another study found that hypocretin neurons in the hypothalamus were activated following exposure to a traumatic event in mice and this activation was associated with increased anxiety-like behavior (39). Our study demonstrated that the blockade of hypocretin receptors in BLA reduced MPS-induced freezing behavior, suggesting that the hypocretin system was involved in the development of PTSD symptoms following trauma exposure. Furthermore, a recent study found that hypocretin signaling in the amygdala was altered in rats with PTSD. The study found that c-fos expression levels in the lateral hypothalamus are higher in PTSD rats than that in naïve rats (29). Furthermore, the expression of hypocretin receptor 1 is significantly higher following the fear conditioning when compared with the naïve rats (29). Our current result is consistent with their observations and further proved that the BLA hypocretin signal may be dysfunctional in individuals with PTSD.

While the research on the relationship between hypocretin and the amygdala in PTSD is still in its early stages, these aforementioned findings suggest that the hypocretin system may play a role in regulating the hyperactivity of the amygdala in individuals with PTSD. Dysregulation of the hypocretin system may contribute to the development of PTSD symptoms following trauma exposure. Our study reveals that the hypocretinergic system plays an important role in the acquisition stage of fear memory formation. However, the current findings in our experiment cannot demonstrate whether the hypocretinergic system is involved in fear memory consolidation or extinction of PTSD mice. Therefore, further studies and experiments are required to reveal the role of hypocretin in PTSD. Meanwhile, it is also important to note that while the amygdala and the hypocretin system have been identified as potential targets for the treatment of PTSD, the complex nature of PTSD suggests that a multifaceted approach to treatment may be necessary. Therapy, medication, and other interventions may be required to address the various aspects of the disorder.

Lemborexant and suvorexant are dual hypocretin receptor antagonists that are mainly used in adult insomnia medication in clinics. However, the hypocretinergic system is not a common therapeutic target in other neurological diseases. Meanwhile, TCS1102 derivatives, specifically in relation to hypocretin modulation, have not been extensively studied or reported in the literature. TCS1102 itself is a specific hypocretin dual receptor antagonist, and while research has focused on developing and studying hypocretin antagonists for potential therapeutic applications, derivatives of TCS1102 or closely related natural compounds have not been widely explored. Only a few hypocretin antagonists derived from the natural compound have been discovered in recent years (40). It is conceivable that in the future, researchers may pursue the purification and analysis of compounds related to TCS1102 or explore natural products for their potential effects on hypocretin modulation or related pathways. These investigations could aim to identify compounds with similar or improved effects on hypocretin receptors, potentially leading to the development of novel therapeutic agents for disorders involving hypocretin dysregulation.

In this study, we discovered that hypocretin in the basolateral amygdala (BLA) plays a role in regulating stress and anxiety behaviors observed in mice with PTSD induced by MPS. We achieved this by using pharmacological blockade. However, it is important to note that there is a reciprocal connection between the BLA and the lateral hypothalamic area (LHA). Our findings only suggest that the transmission direction from the LHA to BLA may be involved in the exhibited PTSD behaviors, rather than the projection direction from BLA to LHA. Moreover, the significance of these reciprocal projections during PTSD remains unclear. In future studies, we plan to simultaneously record local field potentials between the BLA and LHA and analyze coherence and Granger causality to better understand the transmission direction.

In conclusion, the relationship between hypocretin and the amygdala in PTSD is an area of active research, with findings suggesting that the hypocretin system may play a role in regulating the hyperactivity of the amygdala in individuals with PTSD. Further research is needed to fully understand the mechanisms underlying the relationship between these two systems and to develop effective treatments for PTSD. Our results indicated that the MPS protocol could be used as a potential mouse PTSD-like behavior model in fear memory studies, and the hypocretin receptors located in BLA might be important for the fear memory formation in the MPS-induced PTSD model.

## 5. Conclusion

In this study, our research reveals that the MPS protocol can effectively induce fear responses in mice, which mimics the PTSD intrusion symptom in humans. Based on this MPS-induced PTSD-like model, we studied the role of the hypocretin signal in BLA. Our data suggested that the BLA orexin signal might have partial effects in relieving the MPS-induced fear response and anxiety-like behavior. However, additional research is still necessary to validate and understand the fully underlying mechanisms of hypocretin in PTSD-like animals.

## Data availability statement

The original contributions presented in the study are included in the article/supplementary material, further inquiries can be directed to the corresponding authors.

## Ethics statement

The animal study was reviewed and approved by Institutional Animal Care and Use Committee (IACUC) of National Taiwan University.

## Author contributions

T-YL performed experiments, data collection, data analysis, and draft writing. P-LY contributed to the experimental designs, data interpretation, and manuscript writing. F-CC contributed to the hypothesis establishment, experimental designs, data interpretation, and manuscript writing. All authors contributed to the article and approved the submitted version.

## Funding

This work was supported by the Ministry of Science and Technology, ROC, grant 105-2320-B-002-059-MY3, 111-2320-B-002-040-, and National Taiwan University grant 112L892301.

## Conflict of interest

The authors declare that the research was conducted in the absence of any commercial or financial relationships that could be construed as a potential conflict of interest.

## Publisher’s note

All claims expressed in this article are solely those of the authors and do not necessarily represent those of their affiliated organizations, or those of the publisher, the editors and the reviewers. Any product that may be evaluated in this article, or claim that may be made by its manufacturer, is not guaranteed or endorsed by the publisher.

## References

[1] American Psychiatric Association. Diagnostic and statistical manual of mental disorders: DSM-5. 5th ed. Washington, D.C: American Psychiatric Association (2013). 947 p.

[2] Bryant RA. Post-traumatic stress disorder: a state-of-the-art review of evidence and challenges. World Psychiatry. (2019) 18:259–69. doi: 10.1002/wps.20656, PMID: 31496089 PMC6732680

[3] Sareen J. Posttraumatic stress disorder in adults: impact, comorbidity, risk factors, and treatment. Can J Psychiatr. (2014) 59:460–7. doi: 10.1177/070674371405900902, PMID: 25565692 PMC4168808

[4] Ehlers A Hackmann A Michael T. Intrusive re-experiencing in post-traumatic stress disorder: phenomenology, theory, and therapy. Memory. (2004) 12:403–15. doi: 10.1080/09658210444000025, PMID: 15487537

[5] Shalev AY Douglas BJ. Posttraumatic stress disorder: from neurobiology to clinical presentation In: Bremner JD, editor. Posttraumatic Stress Disorder. Hoboken, NJ: John Wiley & Sons, Ltd (2016). 1–26.

[6] Morris MC Rao U. Psychobiology of PTSD in the acute aftermath of trauma: integrating research on coping, HPA function and sympathetic nervous system activity. Asian J Psychiatr. (2013) 6:3–21. doi: 10.1016/j.ajp.2012.07.012, PMID: 23380312 PMC3565157

[7] Harvey AG Jones C Schmidt DA. Sleep and posttraumatic stress disorder: a review. Clin Psychol Rev. (2003) 23:377–407. doi: 10.1016/S0272-7358(03)00032-1 12729678

[8] Germain A Buysse DJ Nofzinger E. Sleep-specific mechanisms underlying posttraumatic stress disorder: integrative review and neurobiological hypotheses. Sleep Med Rev. (2008) 12:185–95. doi: 10.1016/j.smrv.2007.09.003, PMID: 17997114 PMC2490669

[9] Kessler RC Sonnega A Bromet E Hughes M Nelson CB. Posttraumatic stress disorder in the National Comorbidity Survey. Arch Gen Psychiatry. (1995) 52:1048–60. doi: 10.1001/archpsyc.1995.03950240066012 7492257

[10] Whiteford HA Degenhardt L Rehm J Baxter AJ Ferrari AJ Erskine HE. Global burden of disease attributable to mental and substance use disorders: findings from the global burden of disease study 2010. Lancet. (2013) 382:1575–86. doi: 10.1016/S0140-6736(13)61611-6, PMID: 23993280

[11] Borghans B. Animal models for posttraumatic stress disorder: an overview of what is used in research. WJP. (2015) 5:387–96. doi: 10.5498/wjp.v5.i4.387, PMID: 26740930 PMC4694552

[12] Flandreau EI Toth M. Animal models of PTSD: a critical review. Curr Top Behav Neurosci. (2017) 38:47–68. doi: 10.1007/7854_2016_65, PMID: 28070873

[13] Deslauriers J Toth M Der-Avakian A Risbrough VB. Current status of animal models of posttraumatic stress disorder: behavioral and biological phenotypes, and future challenges in improving translation. Biol Psychiatry. (2018) 83:895–907. doi: 10.1016/j.biopsych.2017.11.019, PMID: 29338843 PMC6085893

[14] Schöner J Heinz A Endres M Gertz K Kronenberg G. Post-traumatic stress disorder and beyond: an overview of rodent stress models. J Cell Mol Med. (2017) 21:2248–56. doi: 10.1111/jcmm.13161, PMID: 28374949 PMC5618668

[15] Verbitsky A Dopfel D Zhang N. Rodent models of post-traumatic stress disorder: behavioral assessment. Transl Psychiatry. (2020) 10:1–28. doi: 10.1038/s41398-020-0806-x 32376819 PMC7203017

[16] Souza RR Noble LJ McIntyre CK. Using the single prolonged stress model to examine the pathophysiology of PTSD. Front Pharmacol. (2017) 8:615. doi: 10.3389/fphar.2017.00615, PMID: 28955225 PMC5600994

[17] Bourin M Petit-Demoulière B Dhonnchadha BN Hascöet M. Animal models of anxiety in mice. Fundam Clin Pharmacol. (2007) 21:567–74. doi: 10.1111/j.1472-8206.2007.00526.x 18034657

[18] Cryan JF Holmes A. The ascent of mouse: advances in modelling human depression and anxiety. Nat Rev Drug Discov. (2005) 4:775–90. doi: 10.1038/nrd1825, PMID: 16138108

[19] Ehrlich I Humeau Y Grenier F Ciocchi S Herry C Lüthi A. Amygdala inhibitory circuits and the control of fear memory. Neuron. (2009) 62:757–71. doi: 10.1016/j.neuron.2009.05.026, PMID: 19555645

[20] Cain C Sullivan R. Amygdala contributions to fear and safety conditioning: insights into PTSD from an animal model across development In: Bremner JD, editor. Posttraumatic Stress Disorder. Hoboken, NJ: John Wiley & Sons, Ltd (2016). 81–104.

[21] Maren S. The amygdala, synaptic plasticity, and fear memory. Ann N Y Acad Sci. (2003) 985:106–13. doi: 10.1111/j.1749-6632.2003.tb07075.x 12724152

[22] Kandel ER Koester JD Mack SH Siegelbaum SA eds. Emotion In: Principles of Neural Science. 6th ed. New York, NY: McGraw Hill (2021)

[23] Bryant RA Kemp AH Felmingham KL Liddell B Olivieri G Peduto A. Enhanced amygdala and medial prefrontal activation during nonconscious processing of fear in posttraumatic stress disorder: an fMRI study. Hum Brain Mapp. (2008) 29:517–23. doi: 10.1002/hbm.20415, PMID: 17525984 PMC6870569

[24] Rauch SL Whalen PJ Shin LM McInerney SC Macklin ML Lasko NB. Exaggerated amygdala response to masked facial stimuli in posttraumatic stress disorder: a functional MRI study. Biol Psychiatry. (2000) 47:769–76. doi: 10.1016/S0006-3223(00)00828-3, PMID: 10812035

[25] Inutsuka A Yamanaka A. The physiological role of orexin/hypocretin neurons in the regulation of sleep/wakefulness and neuroendocrine functions. Front Endocrinol. (2013) 4:18. doi: 10.3389/fendo.2013.00018 PMC3589707 23508038

[26] Flores Á Saravia R Maldonado R Berrendero F. Orexins and fear: implications for the treatment of anxiety disorders. Trends Neurosci. (2015) 38:550–9. doi: 10.1016/j.tins.2015.06.005, PMID: 26216377

[27] Ohno K Sakurai T. Orexin neuronal circuitry: role in the regulation of sleep and wakefulness. Front Neuroendocrinol. (2008) 29:70–87. doi: 10.1016/j.yfrne.2007.08.001, PMID: 17910982

[28] Soya S Shoji H Hasegawa E Hondo M Miyakawa T Yanagisawa M. Orexin receptor-1 in the locus coeruleus plays an important role in cue-dependent fear memory consolidation. J Neurosci. (2013) 33:14549–57. doi: 10.1523/JNEUROSCI.1130-13.2013, PMID: 24005305 PMC6618384

[29] Salehabadi S Abrari K Elahdadi Salmani M Nasiri M Lashkarbolouki T. Investigating the role of the amygdala orexin receptor 1 in memory acquisition and extinction in a rat model of PTSD. Behav Brain Res. (2020) 384:112455. doi: 10.1016/j.bbr.2019.112455, PMID: 32044404

[30] Sears RM Fink AE Wigestrand MB Farb CR de Lecea L LeDoux JE. Orexin/hypocretin system modulates amygdala-dependent threat learning through the locus coeruleus. Proc Natl Acad Sci. (2013) 110:20260–5. doi: 10.1073/pnas.1320325110, PMID: 24277819 PMC3864341

[31] Curzon P Rustay NR Browman KE. Cued and contextual fear conditioning for rodents In: Buccafusco JJ, editor. Methods of Behavior Analysis in Neuroscience. 2nd ed. Boca Raton (FL): CRC Press/Taylor & Francis (2009) 21204331

[32] Hoerster KD Lai Z Goodrich DE Damschroder LJ Littman AJ Klingaman EA. Weight loss after participation in a national VA weight management program among veterans with or without PTSD. PS. (2014) 65:1385–8. doi: 10.1176/appi.ps.201300404 25123784

[33] LeardMann CA Woodall KA Littman AJ Jacobson IG Boyko EJ Smith B. Post-traumatic stress disorder predicts future weight change in the millennium cohort study. Obesity. (2015) 23:886–92. doi: 10.1002/oby.21025, PMID: 25776806

[34] Lo Y Yi PL Hsiao YT Chang FC. Hypocretin in locus coeruleus and dorsal raphe nucleus mediates inescapable footshock stimulation (IFS)-induced REM sleep alteration. Sleep. (2022) 45:zsab301. doi: 10.1093/sleep/zsab301 34969120

[35] Sakurai T Mieda M. Connectomics of orexin-producing neurons: interface of systems of emotion, energy homeostasis and arousal. Trends Pharmacol Sci. (2011) 32:451–62. doi: 10.1016/j.tips.2011.03.007, PMID: 21565412

[36] Thompson JL Borgland SL. A role for hypocretin/orexin in motivation. Behav Brain Res. (2011) 217:446–53. doi: 10.1016/j.bbr.2010.09.028, PMID: 20920531

[37] Dustrude ET Caliman IF Bernabe CS Fitz SD Grafe LA Bhatnagar S. Orexin depolarizes central amygdala neurons via Orexin receptor 1, phospholipase C and sodium-calcium exchanger and modulates conditioned fear. Front Neurosci. (2018) 12:934. doi: 10.3389/fnins.2018.00934, PMID: 30618563 PMC6305451

[38] LeDoux JE. Emotion circuits in the brain. Annu Rev Neurosci. (2000) 23:155–84. doi: 10.1146/annurev.neuro.23.1.155 10845062

[39] Sharko AC Fadel JR Kaigler KF Wilson MA. Activation of orexin/hypocretin neurons is associated with individual differences in cued fear extinction. Physiol Behav. (2017) 178:93–102. doi: 10.1016/j.physbeh.2016.10.008, PMID: 27746261 PMC5391308

[40] Yamanaka M Iwata H Masuda K Araki M Okuno Y Okamura M. A novel orexin antagonist from a natural plant was discovered using zebrafish behavioural analysis. Eur Rev Med Pharmacol Sci. (2020) 24:5127–39. doi: 10.26355/eurrev_202005_21207, PMID: 32432777

